# Child Well-Being, Family Functioning, and Contextual Strain: A Study of Multi-Assisted Low-Income Families

**DOI:** 10.3390/children11121533

**Published:** 2024-12-18

**Authors:** Paula Cristina Martins, Vítor Hugo Oliveira

**Affiliations:** 1 Centro de Investigação em Estudos da Criança, Universidade do Minho, 4710-057 Braga, Portugal; 2 Centro de Investigação e Inovação em Educação, Instituto Politécnico do Porto, 4200-465 Porto, Portugal; vhpo@outlook.com

**Keywords:** child poverty, conditional cash transfer, parenting, parental stress, welfare

## Abstract

Objectives: This study aimed to examine the effects of poverty on child well-being and family functioning among low-income families. Specifically, it explored the role of parental stress, family dynamics, and contextual strain on children’s behavioral and emotional outcomes. Using a sample of families receiving welfare support, the research sought to assess the impact of conditional cash transfer programs and the involvement of Child Protective Services on child development and family well-being. Methods: This cross-sectional study involved 99 children aged 8–12 from low-income, welfare-assisted families recruited from the Porto Metropolitan Area. Parental stress, parenting attitudes, family functioning, and child outcomes were assessed using standardized questionnaires. Families with and without Child Protective Services involvement were compared, and a cumulative index of contextual strain was developed to measure the multidimensional impact of stressors on child outcomes. Results: The results revealed that 53% of children exhibited clinical or borderline internalizing behaviors, 47% showed externalizing behaviors, and 39% experienced low psychological well-being. High levels of parental stress, low parenting competence, and significant contextual strain were associated with poorer child outcomes. Families involved with Child Protective Services showed no significant differences in parent or family characteristics, but children from these families exhibited fewer behavioral problems. Conclusions: The study highlights the pervasive impact of poverty and contextual strain on child development, emphasizing the need for more comprehensive interventions. Family functioning and parental stress are critical factors influencing child well-being, pointing to the importance of addressing these areas through targeted welfare and support programs to reduce the intergenerational transmission of poverty and improve child outcomes.

## 1. Introduction

Over the last few decades, tackling child poverty has been on the policy agenda at both the European Union (EU) and member state levels [1,2]. In 2023, 24.8% of children in the EU were at risk of poverty or social exclusion, although the rates varied greatly, ranging from 10.7% in Slovenia to 39% in Romania [3]. Children are the population age group at higher risk of poverty or social exclusion in most EU countries, particularly those from single-parent households, with low education, with low work intensity, and from migrant background households [3]. Despite signs of continued convergence in poverty rates between EU countries up to the beginning of the 2008 financial crisis, the social and economic shocks that followed, along with the pandemic, worsened the poorest countries, and divergences in policies and results became evident [4]. High levels of deprivation have been observed despite public investment and the implementation of different programs at national and local levels, many of which are in partnership with NGOs. However, a more in-depth perspective is needed regarding the well-being of children growing up in poverty and the proximal factors that may help or hinder their health and development. As [5] demonstrates, since 2003, the poverty rate among the resident Portuguese population has fluctuated between 15% and 20%. Notably, the child poverty rate (ages 0–17) has consistently exceeded the overall poverty rate. Moreover, the impact of social transfers on reducing child poverty has been very limited.

In addition to the socioeconomic trends, the conditions of child poverty are specific. Children are at a higher risk of poverty and social exclusion than other social groups. Poverty also affects children differently from adults; children depend on their families and communities to promote their well-being. Furthermore, experiencing poverty in childhood is linked to negative developmental effects that increase the likelihood of experiencing poverty in adulthood [6]. Child poverty is an economic and social problem with political, ethical, and developmental repercussions. As [7] claims, it is a matter of social justice as it undermines the exercise of rights by children, compromises the enforcement of states’ obligations, and constitutes a major public health challenge. In Portugal, psychological research in this field remains largely underdeveloped. Notable exceptions include the study by [8], conducted in collaboration with Spain, and the ongoing initiatives by the ProChild Collaborative Laboratory. Using an urban sample of children from low-income families who were recipients of a conditional cash transfer program, the present study focuses on analyzing the links between parental and familial characteristics and child well-being and adjustment outcomes as indicators of the effectiveness of family welfare programs.

### 1.1. Developmental and Ecological Correlates of Child Poverty

Child poverty is consistently linked to higher physical, emotional, and behavioral morbidity in children [7]. These links include worse general health and allostatic load [9,10] and lower levels of health-related quality of life [11]. In addition, experiencing poverty is associated with a higher prevalence of mental health problems, maladaptive behavior, and lower social competence [12,13,14,15], as well as lower cognitive performance, learning disabilities, and academic failure [16,17,18,19].

Compared with more affluent children, poor children are disproportionately exposed to adverse material and psychosocial conditions that often result in suboptimal developmental pathways throughout childhood into adulthood [20,21]. Such adverse conditions or poverty co-factors are intertwined with the everyday lives of low-income families. These include children’s exposure to multiple risk factors in their social ecologies, such as low parental qualifications, occupational insecurity, parental stress, negative parental behavior, and parents’ mental health problems [22,23,24,25,26]. In addition, poor children are more exposed to ecological chaos, including poor living conditions and material deprivation [27,28], familial conflict/disruption, low social support [29], and social isolation in low-resourced neighborhoods [30].

Moreover, children living in poverty are at a higher risk of maltreatment, which, in turn, has deleterious effects on several aspects of their development [31,32,33,34]. Complex pathways between low-income families and child maltreatment have been examined, and additive and interactive effects have often been associated with an increased probability of maltreatment [31]. These include the co-action of factors such as single parenthood, economic hardship, low parental competence and inadequate parent–child relationships, parental mental health problems, substance abuse, domestic violence, family conflict, and low access to social resources [31,35,36,37].

### 1.2. Theoretical Underpinnings

From a theoretical standpoint, the present study was informed by bioecological and social ecology approaches [38,39,40]. The bioecological model proposes that human development occurs through qualitatively changing interactions between a child and elements in their ecology over time. These mutually affecting factors act in a non-linear, spiraling, and dynamic way and emanate from different contexts in a topologically concentric system [38,39]. A developing child with agency, dispositional attributes, and ecological resources may achieve competence or develop dysfunction depending on the quality of proximal processes provided by their developmental contexts [38]. Contexts influence proximal processes and developmental outcomes not only through the resources provided but also through their consistency and stability over time. In the context of deprivation and disorganization, dysfunction is probably more severe and frequent [39,41]. The ecology of child poverty is characterized by higher exposure to adverse conditions and contextual strain, with an abundance of proximal risks and lower developmental support. These conditions are responsible for the proximal processes and experiences that shape children’s lives, well-being, and development. Here, family functioning plays a central role in these processes [40].

As mentioned above, poverty is consistently linked to factors that affect the family ecology: quality of family life, family functioning, and parenting [42]. In addition, several studies have concluded that poverty indirectly affects child well-being through its impact on parenting practices and the parent–child relationship; social and economic hardship may alter and impair the way families provide care and stimulate and respond to children’s needs in a way that is beneficial to their adjustment, also referred to as family economic stress and family process models [24,25,43,44,45]. In addition, inadequate resources and poorer family functioning, including health care, household conditions, stimulation, childcare and education, and parenting practices, seem to compose a complex network of material and psychosocial factors that act cumulatively, explaining, better or worse, cognitive and socioemotional outcomes for poor children [23,29,40,46].

### 1.3. Child Poverty and Social Policy

Social policies that address child poverty are important not only to reduce its prevalence but also to prevent its pervasive negative effects. Among others, policies include social transfers, such as child benefits, family allowances, disability benefits, and unemployment insurance. However, although social transfers are essential for protecting children from poverty and improving their lives (e.g., financial and material security), they are insufficient. European countries have a diverse bundle of cash transfer programs for families, and, as expected, different policy approaches are associated with different results. For example, activation policies for parents, especially mothers (e.g., job training, work incentives, support for maternal employment), improved access to child health and education, and strong social policies (e.g., generous universal benefits) have proven effective in reducing child poverty in Nordic countries [1,47,48]. These improved outcomes are shaped by the complementary nature of the different actions implemented holistically [49].

Conversely, compared to work incentives and universal benefits models, means-tested models are less effective in promoting the transition from welfare to work and family autonomy [1]. In Portugal, one of the main social policies is the “Rendimento Social de Inserção” (RSI), a national program implemented at the local level that aims to reduce the intensity of poverty affecting socially vulnerable and low-income families. It consists of a conditional cash transfer (CCT) provided by Social Security that complements family income, accompanied by a social integration program managed by local NGOs [50]. The program includes interventions such as job training and skill development, adult education, and access to services. Families may also receive other social benefits and allowances. However, even though the educational interventions of this CCT program are theoretically included in regulations, their implementation varies greatly across regions and services, and a national or local assessment of the impact of the RSI on child and family well-being outcomes is scant.

Other models of CCTs are being implemented and evaluated worldwide. Promising results have been found, especially in developing countries with lower baseline indicators, particularly in programs complemented by the provision of basic services targeting child well-being [51]. In high-income countries, CCTs based on behavioral change (e.g., work, health, education) are also being implemented with inconsistent results, as developmental gains for children are not fully established and long-term effects are unknown [45,52]. For example, the Opportunity NYC Family Rewards CCT initiative [53] showed positive economic and material effects for families but small effects on children’s educational and health outcomes, except for previously high-performing adolescents. Conversely, the New Hope Project [45], a more holistic welfare-to-work program that provides cash supplements, childcare, health insurance subsidies, and intensive case management, was effective in terms of family poverty rates, children’s school performance, and externalizing behavior. Despite some successes observed in interventions for childhood care and education and adult activation strategies, these policies and programs have not yet eliminated poverty and its consequences for children [54].

The quality of the environments in which young people grow shapes their well-being and development. Therefore, the study of the resources and processes leading to the positive adaptation of children whose lives are threatened by poverty is warranted [40,55,56]. Studies on child development in stressful environments emphasize the role of both structural inequalities and proximal factors as threats to children. These contextual factors include an array of influences, such as stressful life events impacting families, social support, mental health, quality of parenting, and parental stress [57], whose effects act cumulatively at multiple levels [45,58]. In addition, poverty has a pervasive impact on the well-being of children, affecting their physical, mental, emotional, and behavioral domains equally. Child adjustment difficulties are highly relevant to research because problems co-occur and are inherently intertwined, impacting children’s developmental health trajectories into adolescence and adulthood [59,60]. Moreover, the compartmentalization of child development domains may lead to a limited research scope and fragmented responses; thus, a multidimensional assessment of child well-being is required [59]. A comprehensive and multidimensional assessment of child well-being addresses empirical insufficiencies, providing information about the differential impacts of children’s environments and daily experiences on developmental outcomes. Therefore, in the present study, child outcomes were assessed in a multidimensional way, representing behavioral adjustment, social competence, and psychological and emotional well-being.

Furthermore, being poor increases the odds of experiencing a higher number of risks; however, it does not describe the actual conditions, experiences, and adaptation processes of poor families [40]. It is important to analyze child and family well-being in the context of national- and community-level investments (programs and services) that aim to reduce poverty and prevent its effects. Children’s problems largely stem from proximal features of the family environment that are malleable to positive change [61]. Therefore, knowledge of these features is needed to inform and enhance the multi-level efforts exerted to lessen the impact of poverty [40,56].

### 1.4. Present Study

Despite a large body of research supporting the complex effects of poverty on child development, knowledge of the psychosocial adjustment of Portuguese children and families living in poverty is scarce. Moreover, few studies have compared families involved and not involved with Child Protective Services and recipients of a CCT program in relation to their parental and familial characteristics and child outcomes. In this context, [7] stands out, advocating for the adoption of an ecological systemic perspective in research on the well-being of children, referred to as Child Protective Services (CPS). Furthermore, the authors propose the specificity hypothesis, which posits that distinct risk and protective factors situated within children’s distal and proximal contexts may have varying impacts on different dimensions of their well-being. In summary, they conclude that different contextual and relational factors are significant for distinct outcomes. This holistic and nuanced approach is essential for effective interventions. Research examining the proximal mechanisms through which disadvantage operates has high social value and concrete implications for prevention [62] and is pivotal for policy design and resource allocation, as well as for the more effective implementation of psychosocial interventions. In this context, the present study aimed to evaluate the well-being and adjustment of children from multi-assisted, low-income families on welfare, comparing the characteristics and outcomes of families involved with Child Protective Services to those not involved. In addition, building on an in-depth and comprehensive approach to the ecology of child poverty, this study aimed to analyze the links between family and parental characteristics, the cumulative effect of contextual strain on families, and child outcomes. In light of previous literature, we hypothesized that proximal ecological factors related to parents and families would have both an individual and cumulative impact on child outcomes and that the group of families involved with Child Protective Services would be exposed to a higher level of stressors, which, in turn, would be associated with poorer child outcomes.

## 2. Materials and Methods

### 2.1. Participants

The sample for this research was a convenience sample obtained within the framework of the existing protocol between the Department of Social Security of Porto and the research team to study the evolution of indicators of welfare support measures provided to families with children.

This study was based on a sample of 99 children (52% boys) aged 8–12 years (*M* = 10.07, *SD* = 1.42) attending school (years: *M* = 4.36, *SD* = 1.36), whose parents (93% mothers) had an average age of 36.62 years (*SD* = 6.95). Parents had an average number of school years completed of 6.91 (*SD* = 2.87), and most had educational attainment lower than or equal to the 6th grade (58%). Most adults were unemployed (94%); 54% were single, divorced, or widowed; and the remaining 46% were married or in a civil union. Family size ranged from two to eight elements (*M* = 3.99, *SD* = 1.37), the number of children per household ranged from one to five (*M* = 2.10, *SD* = 1.06), 64% of households had one or two children, and 36% had three children or more. Chronic health conditions affected 26% of children, and 32% of families were followed by Child Protective Services.

### 2.2. Procedures

The Department of Social Security of Porto, the second-largest city in the country, in collaboration with local NGOs, purposively selected families from the Porto District using heterogeneous sampling and the following two criteria: being recipients of the RSI (a conditional cash transfer program directed at families at risk of poverty) and having a child between 8 and 12 years of age. Using an iterative process, caseworkers contacted eligible families and obtained informed consent from parents. Two evaluation moments were scheduled, and data collection occurred within the NGOs’ premises to guarantee accessibility to the participants. Parents responded to an assessment battery by completing questionnaires and standardized scales administered by trained researchers. Of the 100 families that agreed to participate, only 88% (involving a total of 99 children) completed the assessment protocol and were included in this study. The study design and protocol were approved by the University Ethics Committee and the Department of Social Security.

### 2.3. Measures

#### 2.3.1. Demographic Variables

Parents provided demographic data on children, adults, and families, including children’s gender, age, school year, and chronic health condition; parents’ gender, age, marital status, employment status, and educational level; family size; number of children; and the existence of a Child Protection Plan (CPP).

#### 2.3.2. Parental Stress

The Parenting Stress Index (PSI) [63] is a 132-item self-report measure based on a multidimensional model of stress that assumes that both the child and parent domain characteristics influence parenting and overall stress. Parents choose from a group of possible answers or indicate the degree to which they agree with each statement, from strongly agree to strongly disagree, using five-point Likert scales (e.g., “The child disobeys more than other children”; “It’s pleasurable to be a mother/father”). Several PSI scores were included in the analyses: parent-domain stress (α = 0.91, subscales ranging from 0.57 to 0.80) related to parent-specific characteristics originating stress, such as competence, attachment, or partner relationship; child-domain stress (α = 0.89, subscales ranging from 0.60 to 0.72) related to child-specific characteristics originating stress, such as mood, adaptability, or hyperactivity; and life stressors related to the amount of external stressful life events experienced by the family, such as changes in family income, unemployment, debts, or judicial problems. In addition, the subscales of social isolation (representing parents’ degree of social support) and depression (representing parents’ affective status) from the parent-domain stress scale were also included.

#### 2.3.3. Parenting Attitudes

The AAPI-2 is a 40-item self-report inventory developed by [64] that focuses on parental child-rearing beliefs and attitudes, providing an index of the risk of child abuse and neglect in five areas: expectations of children, empathy towards children’s needs, use of corporal punishment, parent–child family roles, and children’s power and independence. Parents responded to statements related to child-rearing and education using Likert scales from totally agree to disagree (e.g., “Parents needs are more important than children’s needs”; “Parents who love their children should hit them when they misbehave”). An exploratory factor analysis of the Portuguese adaptation of the AAPI-2 was performed; however, the results were inconclusive, as items clustered ambiguously in factors different from the expected theoretical dimensions. In addition, the explained variance of the model was low. Lack of support for the five original dimensions led to a one-factor solution (see [65,66]). Reliability analysis of the scale, including 40 items, indicated that Cronbach’s alpha was adequate for Form A (α = 0.85) and Form B (α = 0.78). Items were averaged and transformed into a 100-point scale, with higher scores reflecting more developmentally adequate parenting attitudes and a lower risk of maltreatment and/or neglect.

#### 2.3.4. Contextual Strain

A composite index of children’s exposure to contextual strain was created using available data regarding parent and family variables associated with stress and adversity. Variables were dichotomized and coded as follows: marital status (married or in a civil union = 0; single, widowed, or divorced = 1), education (higher than the 6th grade = 0; 6th grade or less/low education = 1), existence of a CPP (no CPP = 0; CPP = 1), parenting attitudes (one standard deviation below the mean = 1; remaining scores = 0), and PSI scales and subscales (parent- and child-domain stress, life stressors, social isolation, and depression; above cutoff for clinical stress = 1, and remaining scores = 0). These dichotomized variables were added to compute a cumulative index representing exposure to contextual strain, where a higher score represents higher levels of exposure.

#### 2.3.5. Child Outcomes

The Child Behavior Checklist [67] is a parent self-administered questionnaire comprising 112 items scored on a three-point Likert scale, representing the frequency of behavioral problems in children (e.g., “the child is disobedient at home”; “the child is easily distracted”; “the child screams a lot”). In addition, 11 items related to the adaptive functioning and competence of the child were completed in relation to three developmental contexts: activity participation, social relations, and school performance (e.g., “tasks performed at home”; “school achievement”; “practice of sports”). A score of internalizing (α = 0.85; e.g., somatic complaints, anxiety/depression) and externalizing (α = 0.88; e.g., delinquency, aggression) behaviors was computed, with higher scores representing a higher occurrence of problems. A score for child competence was also computed, with higher scores representing better levels of social competence in several domains (e.g., academic and social).

Additionally, children’s psychological and emotional well-being was assessed. The parent version of the KIDSCREEN-52 is a self-reported 52-item measure using Likert scales (e.g., “never” to “always”), designed to assess children’s health and well-being [68]. It is organized around 10 dimensions of children’s health-related quality of life (e.g., physical well-being, self-perception, school environment, and social acceptance). In this study, the dimensions (6 items; α = 0.85) (e.g., “has your child felt satisfied with his/her life?”; “has your child have fun?”) and moods and emotions (7 items; α = 0.85) (e.g., “has your child felt alone?”; “has your child felt under pressure?”) were used as indicators of mental health (psychological well-being and moods and emotions), with higher scores representing a better perception of children’s health-related quality of life. Scores were computed by averaging the items in each dimension and transforming the means into a 100-point scale.

### 2.4. Data Analysis

Statistical analyses were performed using IBM SPSS and AMOS 29.0. Patterns of missing values were investigated. The results indicated that the proportion of missing values ranged from 0% to 14%, with an overall average of 5.5%. Missing values were not MCAR (Little’s MCAR test, *χ*2 (1, 72) = 93.98, *p* < 0.05). Consequently, a multiple imputation procedure was implemented with five imputations (10 iterations each). The descriptive and correlation results reported herein are derived from the original and pooled data (see Table 1). Subsequently, frequencies, proportions, means, and standard deviations were calculated to examine the sample characteristics and properties of the variables. The dependent variables of psychological well-being and moods and emotions were not normally distributed and were log-transformed and standardized.

Correlation coefficients were calculated to examine the relationships among the study variables according to their measurement characteristics (Pearson correlation coefficient, Spearman’s rho, and Cramer’s V). To examine the effects of the predictors (parent- and family-level variables and contextual strain) on child outcomes, standardized regression weights and total variance were calculated. The full information maximum-likelihood (FIML) model, which estimates parameters using the available information in the dataset, was used to manage missing data. In Model 1, a set of multiple linear regressions was performed for each dependent variable, in which all predictor variables were entered, thus controlling for individual variable effects. In regression Model 2, the cumulative effect of the contextual strain index was tested for each child adjustment outcome.

## 3. Results

The instruments’ standardized cutoff values were used to describe child outcomes. It was observed that 53% of the children were at the borderline or clinical level for internalizing behaviors and 47% for externalizing behaviors. The remaining children presented behavioral symptomatology at levels considered normal. These outcomes were complemented by the fact that 43% presented low social competence at the borderline or clinical level. Finally, 39% of the children experienced very low levels of psychological well-being, and 44% experienced very low levels of moods and emotions (one standard deviation below the mean). These results indicate a moderate to high developmental risk for the children in the sample.

In terms of parent and family characteristics, a large proportion of parents were single (54%) and had educational attainment equal to or lower than the 6th grade (58%). Thirty-two percent of the families were involved in Child Protective Services with a Child Protection Plan (CPP). More than one-third of parents (34%) were at a higher risk for child maltreatment or neglect, as they presented very low levels in the parenting attitudes variable (one standard deviation below the mean). Parents also reported high levels of stress (above the clinical cutoff) in parent-domain stress (25%), child-domain stress (23%), vertical life stressors (70%), social isolation (15%), and depression (15%). In addition, a large proportion of children and their families experienced higher levels of contextual strain, 26% of whom had more than four stressors. These results indicate a considerable level of risk threatening family functioning and child well-being.

### 3.1. Correlation Results

The bivariate relationships between the child, parent, and family variables are presented in Table 1. Child age and gender and family size were not correlated with the remaining study variables and were excluded from further analyses. Statistically significant correlations emerged, with the marital status being related to having a CPP and life stressors. In addition, parental education was related to parenting attitudes, child-domain stress, and externalizing behaviors in the expected directions (poorer results for lower levels of education), but the correlation was negative for psychological well-being. Having a CPP was also associated with higher contextual strain and poorer social competence in children. In addition, the parenting attitudes score was related to parent child-domain stress, externalizing behaviors, and social competence, with worse results for lower levels of parenting attitudes. Parental stress, particularly child-domain stress, was associated with poorer overall outcomes for children. Similarly, life stressors were correlated with parental depression, and parents’ social isolation and depression were linked to poor child adjustment. As expected, the contextual strain index was linked to all parent- and family-level variables (i.e., variables that contributed to its calculation). Contextual strain was also correlated with children’s internalizing and externalizing behaviors and social competence, with higher levels of contextual strain being associated with poorer overall outcomes.

### 3.2. Parent- and Family-Level Predictors of Child Outcomes

The results of the simple and multiple linear regression analyses predicting child adjustment outcomes from the parent- and family-level variables and contextual strain are shown in Table 2. Regression Model 1, which included all predictor variables for each child outcome, explained moderate levels of variance in internalizing behaviors (37%), externalizing behaviors (52%), social competence (28%), psychological well-being (33%), and moods and emotions (29%). In this model, marital status (single parent) predicted lower levels of moods and emotions, higher parental education predicted lower levels of psychological well-being, and having a CPP was associated with lower levels of externalizing behaviors. Parent-domain stress was associated with lower levels of moods and emotions, and the child-domain stress score was a strong predictor of all child outcomes (lower levels of child well-being and adjustment). Life stressors predicted higher levels of internalizing behaviors, and lower social support (higher isolation reported by parents) predicted worse results in children’s social competence and psychological well-being. Contrary to expectations, parental depressive affect was associated with higher levels of moods and emotions, as reported by parents. Additionally, the parenting attitudes variable lost its predictive power when controlling for the remaining variables in the model. Finally, regression Model 2 (see Table 2), which tested the cumulative effects of the contextual strain index on child outcomes, indicated that higher levels of strain predicted greater behavioral problems (internalizing and externalizing) and lower social competence; however, no associations were found for the remaining child outcomes.

## 4. Discussion

Building on the concept that proximal factors are relevant explainers of the association between poverty and poor developmental outcomes for children, this study focused on examining the associations between parent and family variables and contextual strain on children’s developmental adjustment among a sample of low-income families on welfare and CCT program recipients. Overall, it was observed that (i) a large proportion of children faced several adjustment difficulties and experienced low levels of well-being; (ii) parent and family factors and cumulative contextual strain had a moderate to high impact on children’s overall outcomes; (iii) family functioning was challenged by multiple stressors and risks related to parenting and the parent–child relationship; and (iv) families with a CPP did not differ in terms of parent and family characteristics (except for marital status), but the children from this group showed lower levels of problem behaviors.

Moderate to high developmental risk was found, as a large proportion of children in the sample experienced behavioral, emotional, and social difficulties. These findings confirm the expected deleterious effects of poverty on children’s well-being and warrant more effective interventions that consider the developmental needs of the young. Preventing the deleterious effects of poverty is the first step in tackling the transmission of poverty to adulthood and protecting children’s rights and well-being [7,21]. Our main findings also indicate that parent and family factors, as well as contextual strain, have a moderate to high impact on children’s adjustment. Poorer child outcomes were linked with parents’ low educational attainment, impaired parenting attitudes, parental stress, life stressors, low social support, and higher contextual strain. These pervasive effects were observed not only in relation to internalizing and externalizing symptomatology but also in relation to children’s social competence and psychological well-being.

This study adds to existing research by indicating that the influence of poverty on child well-being operates through proximal processes related to low-resourced families and parental psychological distress [46,69]. In addition, a cumulative effect of contextual strain was observed. Poverty is frequently related to proximal stressors, which build on one another. This experience of adversity in multiple domains of family life has been associated with dysfunctional outcomes for children, partly through its impact on parenting practices and the quality of the home environment [70,71]. Altogether, even though the families in our sample were CCT program recipients for years, as well as the object of intervention/treatment by local services and NGOs, a large proportion of children presented adjustment difficulties associated with malleable variables of parental and family functioning. This raises a question regarding the effectiveness of the programs and services designed and implemented to support these families.

In addition, when controlling for the remaining variables in the regression models, parental stress and parents’ social support were more strongly associated with child outcomes. A robust link was observed between child-domain stress and child adjustment, particularly internalizing and externalizing behaviors and psychological well-being, indicating a possible transactional effect between stressful, inadequate parenting and behavioral difficulties in children, exacerbating each other [57,72]. Adults’ social isolation (lower social support) also constitutes a serious risk for children’s adjustment and is associated with poorer functioning. Studies have found that social support is an important factor in reducing parental stress and ineffective parenting, as well as in enhancing family and child well-being [73,74,75]. This indicates the importance of developing parents’ social relationships as well as parenting competencies and resources, especially for those facing economic hardship, and the importance of enhancing the contribution of significant others outside the household to improve child well-being.

As expected, family functioning was challenged by multiple stressors and difficulties, which could build on one another. A large proportion of parents had to handle single parenthood and low education, were involved with Child Protective Services, and were at risk for child maltreatment or neglect. Several parents experienced stress related to the parent–child relationship and high levels of external life stressors. Some also reported experiencing social isolation and depressive symptoms, which were associated with higher levels of overall stress. Conversely, we found that higher levels of education and better levels of parenting attitudes were associated with lower levels of parental stress. Extant research has recognized the different needs and profiles of multi-assisted families and the challenges faced by services in preventing poorer outcomes for both children and parents in low-income situations [76,77].

We also aimed to compare families on welfare with and without CPP in terms of their characteristics, functioning, and child outcomes, and regarding this issue, our expectations were only partly supported. Unlike previous research, the educational attainment of adults, parenting attitudes, parental stress, social support, and mental health problems did not distinguish between maltreating and non-maltreating low-income families [37,74,78]. However, it was observed that single-parent status and contextual strain were associated with a CPP. As discussed by Fong [35], multiple adversities related to poverty are often associated with families’ involvement with welfare agencies and Child Protective Services. The lack of an overall association between a CPP and family functioning in our study is probably linked to the fact that families were being supported by several agencies for some years, and treatment effects were taking place. The existence of a CPP had a deleterious impact on children’s social and emotional competence, indicating that children with a CPP had lower levels of daily participation in social activities and lower academic performance. However, it was expected that a previous family maltreatment status would have a more pervasive and negative effect on child outcomes (see [79]). In addition, a protective effect was observed for internalizing and externalizing behaviors when the remaining predictors were considered in the model. On the one hand, this could be the result of the intervention of Child Protective Services that helped improve parental functioning and child behavior; on the other hand, this could be due to parental bias and underreporting of behavioral problems of children being supervised by welfare services.

Some results were unexpected, which made the interpretation of the findings challenging. Although higher levels of life stressors were associated with higher levels of internalizing symptoms, they were also associated with higher levels of psychological well-being (the effect was small). This is contrary to expectations and previous research reporting an overall negative impact of stressors on child outcomes [62]. Our results suggest that life stressors influence parental functioning and other family processes and that past stressful events impact children’s behavior; however, parents could be acknowledging the better psychological functioning of the child in the present. In addition, a negative relationship between parental education and children’s psychological well-being could be explained by parents with higher levels of education making a more nuanced evaluation of their children’s psychological functioning. Finally, higher levels of adult depressive affect were associated with higher levels of moods and emotions (as reported by parents), suggesting that parents with higher depressive symptoms could have biased perceptions of their children’s emotional states.

Previous research upholds the idea that program outcomes are better when the support provided to low-income families is broad. Programs that address multiple domains of child and family well-being, as well as policies that directly invest in children, hold promise for higher long-term effectiveness. Comprehensive multiservice interventions best represent the multidimensionality of children’s and family’s needs and well-being in the context of poverty. For these reasons, the main goal of national and local policies should be the promotion of children’s and families’ adjustment, shifting from the categorical services framework to a more comprehensive framework [80]. Experimental evidence suggests that, in addition to cash transfer programs and accessorial poverty reduction policies, investment in the quality of family processes may enhance the effectiveness of the policies implemented, including interventions and services directed at parenting skills, social support, parental mental health, and child health and education [52]. Such programs draw on the notion that preventing the effects of poverty demands intervention on proximal mediating factors to improve family functioning and child adjustment.

These findings should be interpreted in light of several limitations. First, the cross-sectional and correlational design poses an impediment to making any causal inferences. As noted, transactional and cascading effects between child and parent behavior, as well as causal relationships between adverse social ecology and children’s developmental adjustment, could only be established through quasi-experimental or longitudinal designs. However, our findings are consistent with previous research and support many of the expected effect directions. Second, the sampling process and sample size also limit the generalizability of the results beyond the city-based population from which they were derived. Family studies on sensitive topics with hard-to-reach populations are known to encounter challenges related to protocol completion and attrition. Third, low parental literacy, particularly within vulnerable populations, is a well-recognized limitation in this field of research, as it may introduce biases into the findings. Future research should consider employing data triangulation through multiple informants, incorporating observational methods, and adapting data collection tools to address this limitation.

## 5. Conclusions

Childhood poverty is a social problem that gathers diagnostic consensus and raises social concerns but very little effective action. Although always included as an ever-present goal in government programs, reducing child poverty is not an effective political priority and remains a silent and persistent problem. Our study adds to previous research by closely examining the factors that pose a risk to child well-being and family functioning in the context of multi-assisted families and welfare recipients. The findings of this study illuminate the needs, strengths, and resources that may be put to work to improve child and family outcomes and the effectiveness of social policy and intervention. In light of recent studies, families should have adequate income complemented by social transfers and effective activation policies that increase parental attachment to work. In addition, access to essential community-based and in-context services that target child and family well-being in multiple dimensions is essential for effectively addressing the negative effects of poverty. Additionally, an anti-poverty policy should be simple, user-friendly, engaging, non-stigmatizing, and consistently coordinated throughout the family life cycle. As a result, multisystemic and tailored interventions should be designed to effectively reach the target group and phase out gradually as families’ financial and social resources become sustainable and family and child well-being is achieved.

## Figures and Tables

**Table 1:** Correlations, means, standard deviations, and ranges of the study variables.

	1	2	3	4	5	6	7	8	9	10	11	12	13	14	15
1. Marital status	―														
2. Parent education (yrs.)	−0.09	―													
3. Child Protection Plan	0.43 **	−0.14	―												
4. Parenting attitudes	0.02	**0** .45 **	−0.13	―											
5. Parent-domain stress	−0.01	−0.09	−0.04	−0.23 *	―										
6. Child-domain stress	0.06	−0.22 *	0.19	−0.34 **	**0** .52 **	―									
7. Life stressors	**0** .20 *	−0.03	0.18	0.06	0.18	0.17	―								
8. Social isolation	0.15	−0.01	0.03	−0.17	**0** .52 **	0.25	0.13	―							
9. Depression	0.01	−0.09	−0.05	−0.18	**0** .65 **	**0** .38 **	**0** .23 *	**0** .52 **	―						
10. Contextual strain	**0** .46 **	−0.49 **	0.50 **	−0.40 **	**0** .51 **	**0** .52 **	**0** .43 **	**0** .35 **	**0** .37 **	―					
11. Internalizing	−0.04	−0.20	−0.10	−0.26	0.26	**0** .42 **	0.21	**0** .24 *	0.22	**0** .26 *	―				
12. Externalizing	0.06	−0.25 *	−0.01	−0.38 **	**0** .37 **	**0** .64 **	0.11	**0** .27 *	**0** .31 **	**0** .37 **	**0** .45 **	―			
13. Social competence	−0.12	0.18	−0.26*	**0** .30 **	−0.16	−0.36 **	0.05	−0.30 **	−0.14	−0.31 **	−0.22 *	−0.19	―		
14. Psychological WB	−0.13	−0.22 *	−0.03	0.03	−0.20	−0.34 **	−0.08	−0.26 *	−0.13	0.05	−0.25 *	−0.17	0.20	―	
15. Moods and emotions	−0.13	0.11	−0.08	**0** .27 **	−0.25 **	−0.43 **	0.01	−0.14	−0.07	−0.19	−0.37 *	−0.32 **	0.14	**0** .35 **	―
*M*	0.54	6.91	0.32	61.66	127.65	107.79	26.10	14.03	22.40	3.30	59.85	57.49	42.51	86.08	84.81
*SD*	0.50	2.87	0.47	7.47	24.46	22.71	11.98	3.85	6.07	1.80	8.99	9.47	10.81	10.45	13.10
Range	0–1	1–18	0–1	41–84	66–209	57–176	0–55	6–27	9–43	0–9	33–83	33–77	17–67	58–100	40–100

Note. * *p* < 0.05, ** *p* < 0.01. Statistically significant correlations in bold. Range values rounded to the unit.

**Table 2:** Standardized regression weights and explained variance for parent- and family-level predictors of child well-being.

	Internalizing Behaviours	Externalizing Behaviours	Social Competence	Psychological Well-Being	Moods and Emotions
**Model 1 (all predictors)**					
Marital status	0.00	0.06	0.01	–0.19	**–0** .26 *
Parent education (years)	–0.07	–0.06	0.06	**–0** .33 ***	–0.10
Child Protection Plan	**–0** .30 **	–0.27 **	–0.20	0.14	0.12
Parenting attitudes	–0.18	–0.16	0.14	0.02	0.18
Parent-domain stress	–0.18	–0.06	0.18	0.10	**–0** .35 *
Child-domain stress	0.43 ***	0.63 ***	**–0** .32 **	**–0** .44 ***	**–0** .34 **
Life stressors	0.27 **	0.04	0.14	0.13	0.05
Social isolation	0.20	0.07	**–0** .30 *	**–0.** 29 *	0.02
Depression	0.04	0.01	0.03	0.11	0.37 **
*R*^2^	0.37	0.52	0.28	0.32	0.30
**Model 2 (cumulative effects)**					
Contextual strain index	0.29 **	0.38 ***	**–0** .34 ***	–0.02	–0.17
*R*^2^	0.08	0.14	0.12	0.00	0.03

Note. Statistically significant associations in bold followed by explained variance (*R*^2^). * *p* < 0.05, ** *p* < 0.01, *** *p* ≤ 0.001.

## Data Availability

The data presented in this study are available on request from the corresponding author due to their sensitive nature and the legal and ethical limitations determined by the funding institutions and research partners.
